# Physical contact in parent-infant relationship and its effect on fostering a feeling of safety

**DOI:** 10.1016/j.isci.2021.102721

**Published:** 2021-06-12

**Authors:** Sachine Yoshida, Hiromasa Funato

**Affiliations:** 1 Department of Anatomy, Faculty of Medicine, Toho University, Ota-ku, Tokyo 143-8540, Japan; 2 International Institute for Integrative Sleep Medicine (WPI-IIIS), University of Tsukuba, Tsukuba, Ibaraki 305-8575, Japan

**Keywords:** Biological sciences, Neuroscience, Behavioral neuroscience, Sensory neuroscience

## Abstract

The infant-caregiver relationship involves physical contact for feeding, moving, and other cares, and such contact also encourages the infant to form an attachment, an emotional bond with the caregivers. Physical contact always accompanies somatosensory perception, which is detected by mechanosensory neurons and processed in the brain. Physical contact triggers sensorimotor reflexes such as Transport Response in rodent infants, and calm human infants while being carried. Tactile sensation and deep pressure in physical interactions, such as hugging, can function as emotional communication between infant and caregiver, which can alter the behavior and mood of both the infant and caregiver. This review summarizes the findings related to physical contact between the infant and the caregiver in terms of pleasant, noxious, and neutral somatosensation and discusses how somatosensory perceptions foster a feeling of safety that is important for infant's psychosocial development.

## Graphical abstract

**Figure:**
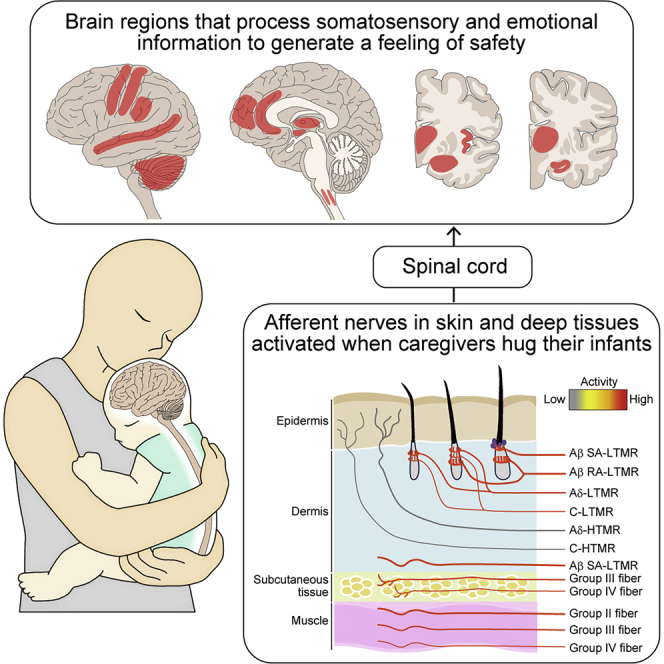

All mammalian infants are fed milk produced by the mother's mammary glands. However, brain maturity and motor ability vary among mammals at the time of birth (Weisbecker and Goswami, 2010). Mice are altricial species that cannot move by themselves, and they need to be fed and taken care of by their mother for the first 3 weeks of their life. Likewise, humans are altricial and take a year after birth to be able to walk. Giraffes, on the other hand, are precocial species that are born very mature and begin walking within an hour of birth (Scheiber et al., 2017). Even in precocial species, young animals are weak; thus they live together with their mothers. Regardless of whether they are altricial or precocial species, all mammals are fed milk from their mothers for a certain period, and during this time, they have close contact with their mothers. Even in aquatic mammal dolphins, the calves stay close to the mothers sides in a specific mother-calf position similar to carrying (Noren, 2008), which helps the calf swim easily within the water currents created by the mother and to take milk from her. Thus, young mammals commonly have close physical contact with mothers for feeding, moving, and/or being safe. Such physical contact and the accompanying somatosensory perception serve as a form of nonverbal communication and are particularly important in the psychophysiological development of human infants, who form an emotional bond with their caregivers, which is called attachment (Bowlby, 1951, 1969; Moriceau and Sullivan, 2005; Sullivan et al., 2011). Indeed, mechanosensation from the skin is the earliest developing sensory modality (Bremner and Spence, 2017; Gallace and Spence, 2010). Human neonates exhibited activation of the postcentral gyrus in response to palm stimulation (Arichi et al., 2012) and could discriminate between touch and pain (Fabrizi et al., 2011). Thus, infants can perceive somatosensory sensation during physical interaction with caregivers, which may shape relationship with caregivers and affect infants' emotional and social development.

Recent advances in the understanding of the somatosensory system enable us to discuss the infant-caregiver relationship in terms of the pleasant perception of touch and hugs. In this review, we first summarize mechanoreceptors and somatosensory modalities that can be active during physical contact between the infant and caregiver. Then, we discuss the physical contact between the infant and the caregiver in terms of different somatosensory perceptions, such as (1) sensory information that is inherently positive and rewarding, (2) painful or noxious sensory stimuli, and (3) neutral sensory information, especially during hugging. Underlying these somatosensory perceptions is cognitive, social, and neurological development of infants. Next, we mention the immediate response to somatosensory input, such as Transport Response, and the long-term effects of the lack of intimate physical contacts during infancy. Finally, we briefly discuss the effect of physical contact with infants on caregivers.

## Somatosensory and pain perception during physical contact

**Figure 1:**
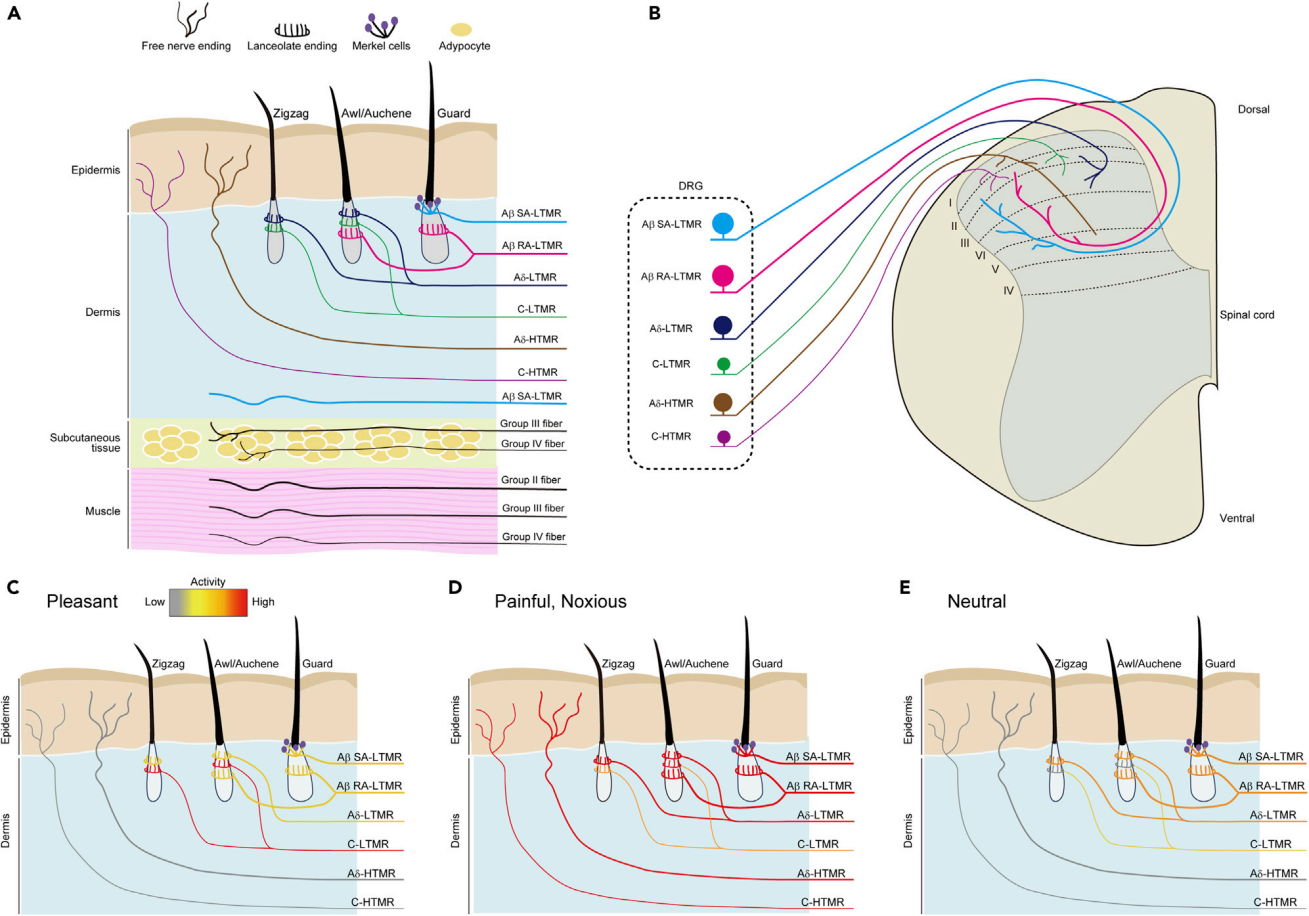
Differential responses of sensory neurons to mechanical stimuli (A) Schematic drawing of cutaneous and muscle afferents in hairy skin. Innocuous mechanical stimuli are detected by nerve endings that surround hairs. In mice, three types of hairs are defined as guard, awl/auchene, and zigzag. Guard hairs are innervated by Aβ SAI-LTMR with Merkel cells (touch domes) and by Aβ RAI-LTMR with lanceolate endings. Awl/auchene hairs are innervated by Aβ-LTMR, Aδ-LTMR, and C-LTMR with lanceolate endings. Zigzag hairs are innervated by both Aδ-LTMR and C-LTMR lanceolate endings. Noxious touch is detected by Aδ-HTMR and C-HTMR that have free nerve endings in the epidermis. The ending structure of Aβ SA-LTMR in deep dermis is unclear. There are sensory afferents in subcutaneous tissues and muscle tissues. Group II, III, and IV fibers correspond to cutaneous Aβ-, Aδ-, and C-fibers, respectively. The endings of these fibers are generally free nerve ending. (B) Spinal projections of cutaneous afferents. Different subtypes of sensory neurons whose cell bodies reside in the dorsal root ganglia (DRG) extend different classes of nerve fibers. Central branches of sensory neurons terminate in the specific lamina of the spinal cord. Central branches of Aβ SA-LTMR and Aβ RA-LTMR arborize into lamina II to V. Aδ-LTMRs and C-LTMRs terminate in lamina II and III. Aδ-HTMRs terminate in lamina I, II, and V. C-HTMRs terminate in lamina I and II. (C–E) Differential activation of cutaneous somatosensory neurons to pleasant gentle stimuli (C), painful, noxious stimuli (D), and neutral or discriminative stimuli (E). (C) A pleasant gentle touch strongly activates C-LTMR and weakly activates Aβ- and Aδ-LTMR. A pleasant touch does not activate Aδ-HTMR and C-HTMR because the pressure of a pleasant touch is below the threshold of the HTMRs. (D) A painful, noxious stimulus strongly activates Aδ-HTMR and C-HTMR. Aβ-HTMR (not depicted) and C-LTMR also respond to noxious stimuli. (E) A neutral tactile sensation that is not inherently pleasant or noxious is detected by Aβ- and Aδ-LTMR. C-LTMR may also be involved. LTMR, low-threshold mechanoreceptor; HTMR, high-threshold mechanoreceptor; SA, slowly adapting; RA, rapid adapting.

Touching and hugging can induce innocuous, pleasant, or noxious sensation through mechanoreceptors in the skin and deeper tissues (Figure 1, Table 1). Innocuous and discriminative tactile sensation is detected by low-threshold mechanoreceptors (LTMRs) such as Aβ-LTMRs, Aδ-LTMRs, and C-LTMRs. These LTMRs innervate hair follicles and form end organs surrounding hair follicles such as lanceolate endings in rodents (Abraira and Ginty, 2013; Li and Ginty, 2014; Zimmerman et al., 2014). Aβ-LTMRs also innervate the Merkel cell-neurite complex or Merkel disks that reside in the basal layer of the epidermis of globous (hairless) skin and hairy skin. The mechanically activated cation channel Piezo2 is expressed in Merkel cells and adjacent Aβ-sensory fibers and is required for mechanical force sensation during touch (Ranade et al., 2014; Woo et al., 2014).

**Table 1:** Mammalian cutaneous mechanosensation

Modality	Optimal stimulus	Skin type	Pressure to be detected	Receptive field	Physiological subtype	Fiber morphology	End organ	Ending location	Markers^a^
Tactile	Skin movement, hair follicle deflection	Glabrous	Low	Small	Aβ RAI-LTMR	Myelinated	Meissner corpuscle	Dermal papillae	Ret, TrkC NFH, Npy2r, KCNQ4, Piezo2
		Hairy					Lanceolate ending	Hair follicle	
	Skin indentation, hair follicle deflection	Glabrous	Low	Small	Aβ SAI-LTMR	Myelinated	Merkel cell	Epidermal basal layer	Ret, TrkC, NFH, VGlut3, Piezo2
		Hairy					Merkel cell (touch dome)	Hair follicle	
	Vibration	Glabrous	Low	Large	Aβ RAII-LTMR	Myelinated	Pacinian corpuscle	Dermis	Ret, Npy2r, Piezo2
	Stretch	Glabrous	Low	Large	Aβ SAII-LTMR	Myelinated	Ruffini corpuscle^b^	Dermis	NFH, Piezo2?
		Hairy					Unclear	Unclear	
	Skin movement, indentation	Hairy	Low	Large	Aβ-Field LTMR	Myelinated	Circumferential ending	Dermis	NFH, TrkC, Ret, Piezo2?
	Hair follicle deflection	Hairy	Low	Small	Aδ-LTMR	Thin myelinated	Lanceolate ending	Hair follicle	TrkB, Cav3.2, Piezo2
	Hair follicle deflection	Hairy	Low	Small	C-LTMR^c^	Unmyelinated	Lanceolate ending	Hair follicle	Th, VGlut3, Mrgprb4, Piezo2
Pain	Mechanical	Both	High	Small	Aβ-HTMR^d^	Myelinated	Unknown	Dermis	Unknown
	Noxious mechanical	Both	High	Small	Aδ-HTMR	Thin myelinated	Free nerve ending	Epidermis	TrkA, CGRP, NFH
	Noxious mechanical	Both	High	Small	C-HTMR	Unmyelinated	Free nerve ending	Epidermis	TrkA, CGRP, Mrgprd, VGlut3

This table is based primarily on the following references: Abraira and Ginty (2013); Bai et al. (2015); Johnson (2001); Nagi et al. (2019); Roudaut et al. (2012); Usoskin et al. (2015); Zheng et al. (2019); Zimmerman et al. (2014).

HTMR, high-threshold mechanoreceptor; LTMR, low-threshold mechanoreceptor; RA, rapidly adapting; SA, slowly adapting.

a Molecular markers have been reported for mice (Finno et al., 2019; Li et al., 2016; Usoskin et al., 2015; Zheng et al., 2019).

b Ruffini corpuscle structure has been found in human digital skin (Cobo et al., 2020) but has not been reported in rodents (Wellnitz et al., 2010).

c There are C-LTMRs that also detect noxious stimulus (Vallbo et al., 1999).

d Nagi et al. Sci Adv 2019 did not explicitly label with Aβ because the classification of Aβ and Aδ afferents based on conduction velocity is not clear-cut in humans (Nagi et al., 2019).

Humans prefer gentle touches, such as a caress, on hairy skin (Jönsson et al., 2017). That is why this type of touch is also called a pleasant touch, which plays a crucial role in the interpersonal relationship between infants and caregivers, between romantic partners, and between close friends. Although Aβ-LTMR and Aδ-LTMR afferents respond to soft brush stroking (Nagi et al., 2019), unmyelinated C-LTMRs are thought to be primarily responsible for the pleasant sensation of gentle touch (McGlone et al., 2014; Morrison et al., 2010; Olausson et al., 2002) (Figure 1C). The firing rate of C-tactile fibers correlates positively with subjective pleasantness in humans (Löken et al., 2009), and the activation of C-LTMRs is also rewarding for mice (Vrontou et al., 2013). Similar to Aβ sensory neurons, Piezo2 is highly expressed in C-LTMR sensory neurons (Usoskin et al., 2015; Wu et al., 2017).

Somatosensory signals are conducted from peripheral tissues to the spinal cord via nerve fibers of sensory neurons whose cell bodies exist in dorsal root ganglia (DRG) and cranial sensory ganglia (Jenkins and Lumpkin, 2017) (Figure 1B). DRG sensory neurons are classified into more than 10 subtypes depending on gene expression and the stimuli that activate them (Finno et al., 2019; Li et al., 2016; Usoskin et al., 2015; Zheng et al., 2019). Each sensory neuron subtype has a characteristic and a graded sensitivity for different stimulus modalities, such as touch, pain, itch, temperature, and chemicals (Zheng et al., 2019). A DRG neuron population characterized by tyrosine hydroxylase corresponds to C-LTMRs (Li et al., 2011; Usoskin et al., 2015).

In addition to afferent fibers from the skin, DRG neurons have afferents from deeper tissues such as subcutaneous tissues and muscle tissues (Hoheisel et al., 2005; Tesarz et al., 2011) (Figure 1A). Muscle afferents are classified into groups I–IV according to conduction velocity and myelination. Group II fibers correspond to cutaneous Aβ fibers, group III to cutaneous Aδ fibers, and group IV to cutaneous C fibers. Nearly half of group IV muscle afferents are LTMRs and not nociceptive (Hoheisel et al., 2005). Single-cell qPCR showed that the molecular profile of DRG neurons that have cutaneous afferents is very similar to that of DRG neurons that have muscle afferents (Adelman et al., 2019). These findings indicate that deep tissue afferents are able to transmit various sensory information similar to the skin. Indeed, Aβ sensory fibers in deep tissues are required for the detection of innocuous and pleasant pressure sensations in which Piezo2 is not involved (Case et al., 2021). The deep pressure generated by compression sleeves, which is independent of C-tactile afferents, provides a subjective pleasant sensation and activates brain regions, including the middle insula and supramarginal gyrus, which partly overlap with but are distinct from brain regions activated by C-tactile stroking (Case et al., 2020).

In contrast to innocuous sensation, noxious touches are mainly detected by high-threshold mechanoreceptors (HTMRs) and nociceptors (Abraira and Ginty, 2013; Hill and Bautista, 2020; Peirs and Seal, 2016) (Figure 1D, Table 1). Nociceptive Aδ- and C-sensory fibers have specialized free nerve endings to detect potentially damaging stimuli and are characterized by the expression of calcitonin gene-related peptide (CGRP) (Usoskin et al., 2015; Zheng et al., 2019). Aβ-HTMRs are also involved in the fast sensation of pain in humans and rodents (Nagi et al., 2019; Peirs and Seal, 2016), although optogenetic activation of Vglut1-positive Aβ-HTMR did not induce aversive reaction (Chamessian et al., 2019). Importantly, C-fibers with a low threshold that are thought to be CGRP negative also exhibit a robust response to noxious mechanical stimuli (Vallbo et al., 1999). Consistent with the finding that CGRP-negative A- and C-fibers are involved in noxious sensation, mice with ablation of CGRP-positive DRG neurons maintained response to noxious mechanical stimuli such as tail clip (McCoy et al., 2013).

**Figure 2:**
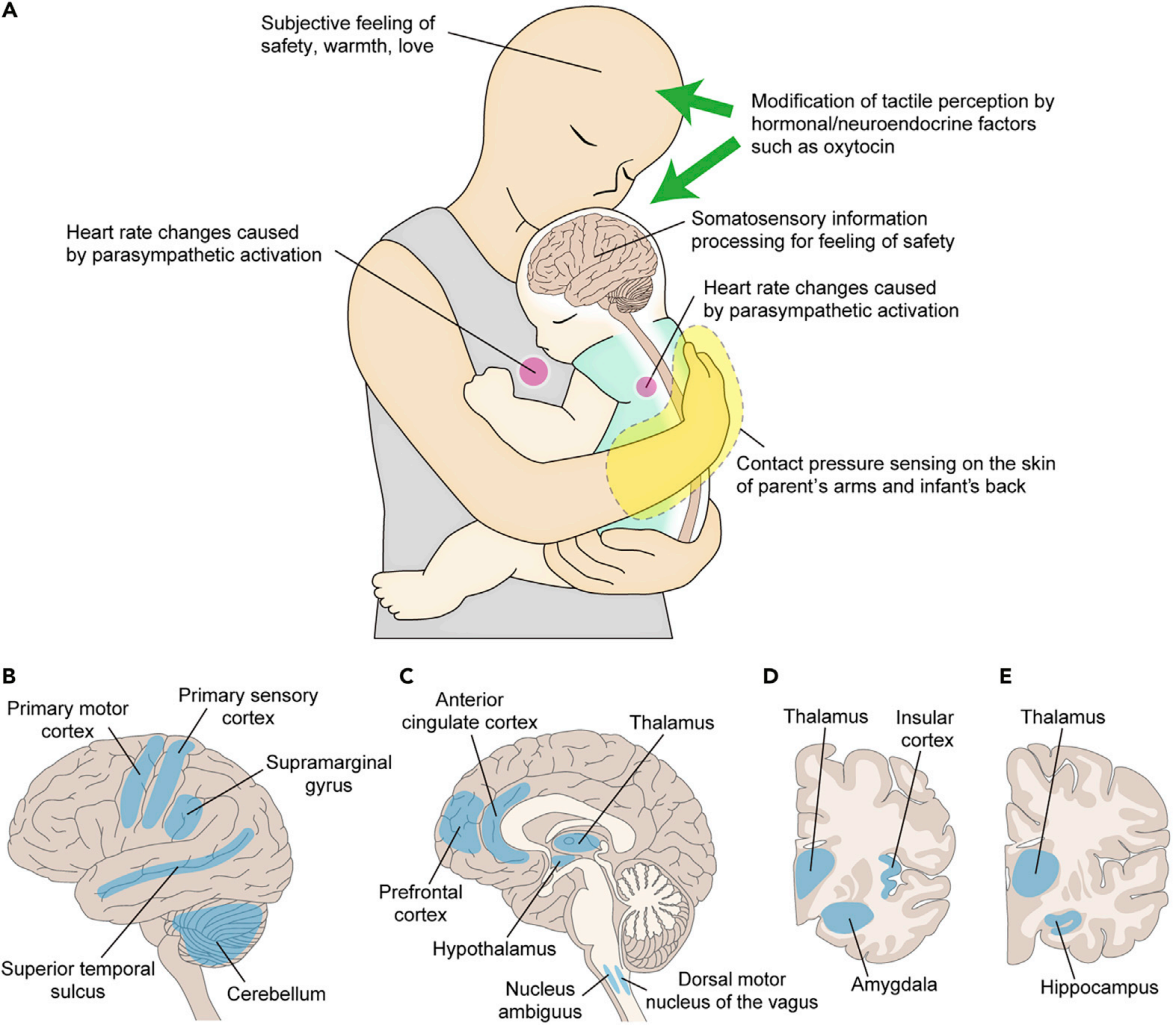
Brain areas processing tactile and affective information during physical contact (A) When the caregiver hugs the infant, the tactile sensation leads to a sense of safety and parasympathetic activation in both the caregiver and the infant. Hormonal and neuroendocrine factors such as oxytocin may also be involved in the modification of sensory perception. (B–E) Brain areas processing tactile and affective information detected by somatosensory neurons during physical contact. Discriminative tactile information is primarily processed in the primary somatosensory cortex, whereas affective touch is processed in the insular cortex. The superior temporal sulcus, anterior cingulate cortex, prefrontal cortex, and amygdala are involved in tactile information processing with reference to emotional and social contexts. Lateral view (B), midsagittal section (C), and coronal section (D,E. D is more rostral to E) of the brain.

The central branches of DRG sensory neurons project to the spinal cord via the dorsal root and, in some cases, the dorsal column nuclei of the brainstem (Abraira and Ginty, 2013; Peirs and Seal, 2016; Todd, 2010). Aβ signals for discriminative touch and pressure information travel primarily along the dorsal column medial lemniscus pathway, are relayed in the ventral posterior nucleus of the thalamus, and reach the primary somatosensory cortex in the postcentral gyrus (Figure 2). C-afferents conveying temperature, itch, and pain terminate in lamina II–III of the spinal cord and then transmit to lamina I neurons from which the spinothalamic tract in the anterolateral funiculus originates. The spinothalamic tract reaches the sensory thalamus after branching to many brainstem neurons, such as the parabrachial nucleus and locus coeruleus. Recent findings suggest that this simple Aβ versus C dual pathway model cannot explain the process of C-LTMR signals for pleasant touch sensation. Rather, the transmission of the pleasant sensation of touch can be processed by both C-LTMR and Aβ-LTMR in an integrated manner, and the C-LTMR signal for pleasant touch may be transmitted via the dorsal column pathway after synaptic connections within the spinal cord (Abraira and Ginty, 2013; Andrew, 2010; Marshall et al., 2019).

After the sensory thalamus, whereas Aβ-LTMR information is transmitted to the primary somatosensory cortex to process fine-grained signals for discriminative touch, C-tactile information is transmitted to and activates the insular cortex, thereby reflecting a positive valence (McGlone et al., 2014; Morrison, 2016; Olausson et al., 2002). The insular cortex has dense fiber connections, receives direct input from the somatosensory thalamus, and processes somatosensory experiences in conjunction with emotional valence and autonomic responses (Eckstein et al., 2020; Gogolla, 2017; Uddin et al., 2017)(Figure 2). In addition, pleasant skin stroking activates the posterior superior temporal sulcus, which correlates with subjective pleasantness (Davidovic et al., 2016). The superior temporal sulcus has projections from the insular cortex and processes the emotional meaning of somatosensory perception in a social context (Schirmer and Adolphs, 2017). The anterior cingulate cortex also shows brush stroke-induced activation correlated with subjective pleasantness (Case et al., 2016). In reality, physical contact is composed of various mechanical forces in terms of duration, degree of pressure, speed, and direction of stroking, which are encoded by a different set of LTMR activities and result in the complex processing of somatosensory perception in the brain regarding emotional valence and social context (Abraira and Ginty, 2013).

The brain has no discrete primary cortical region for pain analogous to the primary somatosensory for discriminative Aβ afferents; rather, noxious stimulation evokes a diffuse pattern of activity in many brain areas, including primary and secondary somatosensory cortices, the insular cortex, anterior cingulate cortex, prefrontal cortex, and amygdala, which largely overlap with the areas for positive emotional processes (Figure 2).

Innocuous and noxious tactile information are not processed independently but can affect each other. For example, slow brushing, which is the optimal stimulus for C-tactile afferents, reduces pain perception from skin heating (Liljencrantz et al., 2017). Thus, during touching and hugging between infants and caregivers, various somatosensory modalities work together to cause a colorful sensation, which helps to form an emotional bond with the caregiver and supports infant care.

Social and interpersonal context may affect somatosensory perception via hormonal and neuroendocrine milieu. A neuropeptide, oxytocin, is involved in social bonding formation and attachment behavior (Feldman, 2017). Oxytocin also affects somatosensory processing and exerts analgesic effects via oxytocin receptors that express somatosensory cortex and DRG sensory neurons (Grinevich and Stoop, 2018), and alters synaptic plasticity in an activity-dependent manner (Pekarek et al., 2020). Nasal administration of oxytocin increased pleasantness of C-LTMR-dependent tactile stimulus (Chen et al., 2020). Thus, the emotional bonding in the infant-caregiver relationship may enhance pleasantness of affective touch and alleviate painful perception (Figure 2A).

## Affective touch and infant care

In the 1950s, Harlow conducted a series of experiments with surrogate mothers that showed how strongly somatosensory comfort attracts rhesus infants (Harlow, 1958; Harlow and Zimmermann, 1959). The infants spent more time with a cloth mother surrogate without a food bottle than with a wire mother surrogate that held a food bottle. Importantly, at anxious and fearful events, the infants clung to a cloth surrogate for feeling secure and used the surrogate as a secure base for exploration. The strong sense of comfort while gently touching them may be one of the reasons why children love fluffy stuffed animals.

In fact, brain imaging studies have demonstrated that infants feel the same gentle touch as adults through the insular cortex. The soft brush stroking of 11- to 36-day-old infants activates the posterior insular cortex, as well as the primary somatosensory cortex (Tuulari et al., 2019). When 2-month-old infants are stroked with a paintbrush, the insular cortex is more strongly activated than the temporal lobe (Jönsson et al., 2018). The gentle and slow stroking optimal for C-tactile afferents of infants reduces brain activity that was induced by noxious stimuli to the skin (Gursul et al., 2018), which suggests the pain-alleviating effect of C-tactile stroking in infants as found in adults (Liljencrantz et al., 2017) and gentle stroking as nonpharmacological management of infant pain. In addition to sensory perception, C-tactile stroking affects the entire body. Gentle stroking at an optimal speed decreases heart rates in preterm infants (Manzotti et al., 2019), suggesting parasympathetic activation similar to adults. Thus, by alleviating noxious perception and enhancing parasympathetic activity, skin stroking helps to nurture the subjective sense of comfort in infants.

As infants grow, the number of brain regions involved in processing affective touch increases. A gentle touch with velvet fabric activates the prefrontal cortex in 10-month-old infants, but not in 3- or 6-month-old infants (Kida and Shinohara, 2013). Functional near-infrared spectroscopy does not detect any reaction specific to affective hand touch or nonaffective touch with a spoon in the superior temporal sulcus area of 5-month-old infants (Pirazzoli et al., 2019). As it matures, this area will respond to maternal touch. Resting-state functional magnetic resonance imaging (fMRI) imaging of 5-year-old children demonstrates that activity in the right posterior superior temporal sulcus and right temporoparietal junction is positively correlated with the frequency of maternal touch (Brauer et al., 2016). As the posterior superior temporal sulcus processes social context (Beauchamp, 2015; Schirmer and Adolphs, 2017), these findings suggest that affective touch from caregivers promotes the functional maturation of brain regions that process social contexts.

Tickling is another somatosensory feeling that has a positive valence for young children accompanied by laughter. The light passive tactile sensation of moving causes a tickling sensation that many children prefer. Similar to human children, young rats prefer the tickling sensation and approach a tickling hand and exhibit ultrasonic vocalization and “joy jumps” (Ishiyama and Brecht, 2016) with which the dopaminergic system is associated (Panksepp and Burgdorf, 2003).

If human infants inherently prefer gentle touches and derive comfort from this sensation, then being deprived of physical contact with their caregivers in the neonatal intensive care unit can be harmful for preterm infants. Indeed, massage therapy in which preterm infants receive slow stroking to compensate for the lack of touch has been repeatedly shown to promote weight gain and cardiac vagal activity in preterm infants (Ferber et al., 2002; Field et al., 2010). Massaging increases the weight gain of preterm infants without changes in their caloric intake (Diego et al., 2005), which may be explained by increased parasympathetic tone and gastric motility for better digestion and absorption of nutrition (Diego et al., 2005). It is also possible that increased parasympathetic activity suppresses the heat production of brown adipose tissues that are rich in infants (Gaspar et al., 2021), which enables infants to better utilize their energy resources for body growth.

Whether mothers or trained professionals provide tactile treatment does not matter for the growth-promoting effect (Ferber et al., 2002). As neonates cannot recognize their parents, it does not matter who provides them with pleasant touches and deep sensations. Moderate pressure, not light pressure, is required for vagal activity and weight gain (Diego et al., 2005), which suggests that deep sensation from subcutaneous and muscle tissues, in addition to gentle touch, plays an important role in these processes.

Kangaroo care was originally developed to keep preterm infants warm and to enhance home care to prevent cross-infection in the hospitals in Bogota, Columbia, to cope with a lack of incubators (Whitelaw and Sleath, 1985). Kangaroo care intervention enables premature infants to maintain their body temperature through skin-to-skin contact with the parent's body. Even today, the World Health Organization recommends continuous Kangaroo maternal care for preterm newborns. Kangaroo care is thought to contribute to the psychological development of preterm infants by promoting emotional stability and the formation of bonding. Premature infants who received the Kangaroo care intervention for 14 days after birth show enhanced physiologic organization and cognitive development at the age of 10 years compared with controls (Feldman et al., 2014), suggesting that the Kangaroo care experience changes the relationship between the infant and the caregiver and has positive long-term effects on the child.

Meany and colleagues directly demonstrated the long-term effects of gentle physical contact with the mother, such as grooming and licking, on rat infant development. When a rat infant is taken away from the mother for 15 min and then brought back to her, the mother rat increases her licking/grooming behavior toward the infant. This postnatal handling shows that the frequency of grooming by the mother can explain differences in resilience to stress and hippocampal glucocorticoid receptor (GR) expression in the offspring after maturation (Francis et al., 1999), which is due to reduced DNA methylation at the GR gene (Weaver et al., 2004). Human infants also show differences in DNA methylation of the GR promotor and salivary cortisol concentrations depending on whether they are breastfed or not during the first 5 months of life (Lester et al., 2018). Although detailed causal relationships remain to be elucidated, these results suggest that nurturing quality can alter gene expression in both rat and human infants.

## Pain and noxious stimuli for infants

Although it is not behaviorally obvious, recent brain activity measurements have shown that neonates experience pain similar to that felt by adults (Fitzgerald, 2005; Verriotis et al., 2016). For example, an fMRI study showed that neonate brain activities evoked by noxious stimulation highly overlap with those seen in adult brains (Goksan et al., 2015). Neonates exhibit nociceptive event-related potentials in response to heel lances (Jones et al., 2017). Nociceptive brain activity is enhanced by stress level, which can be evaluated by salivary cortisol and high-frequency heart rate variability (Jones et al., 2017), suggesting that the nurturing environment alters infants' sensitivity to pain. In fact, an analgesic effect has been observed in human infants and rat pups during non-nutritive sucking (Blass et al., 1995; Mangat et al., 2018). Skin-to-skin contact with caregivers is used for pain management in the neonatal intensive care unit (Bucsea and Pillai Riddell, 2019). In addition to acute analgesic effects, attachment to caregivers has been associated with protection from pain discomfort, and infants experiencing pain show disturbed attachment patterns (Failo et al., 2019).

Somewhat strangely, rat infants seem to be attracted to painful stimuli at a certain period of development. When rat infants were exposed to an odor while being subjected to painful stimuli, they subsequently preferred the odor (Moriceau and Sullivan, 2005). This preference for a pain-associated cue is not due to the pups' inability to feel pain. Such counterintuitive preference learning is regarded as an adaptive response to maintaining proximity to the mother. As rat pups are very immature and need full maternal care before day 9, neonatal rats are confined to the nest, and most sensory stimuli, including pain and noxious stimuli, are provided by the mother. It is feasible that pups can learn only preferences. This biased learning of infant rats may be due to noradrenaline that is released from locus coeruleus neurons, the immaturity of the amygdala for adverse learning, and an attenuated corticosterone release in response to noxious stimuli (Moriceau and Sullivan, 2005). Approximately 6–14 days postpartum, rat infants show only a slight increase in corticosterone in response to stress, and this period is called the “stress hyporesponsive period” (Santiago et al., 2017; Schmidt, 2019). The hypothalamus-pituitary-adrenal (HPA) axis is suppressed and corticosterone levels are low, whereas the amygdala is not activated, and rat infants do not express fear in response to external stressful stimuli. From days 12–16, preference for the pain-conditioned odor is recognized only with maternal presence, but pups can learn to avoid the pain-conditioned odor in the absence of their mother (Moriceau and Sullivan, 2006). Maternal presence suppresses the corticosterone increase to inhibit amygdala-dependent fear learning (Santiago et al., 2017). After postnatal day 16, stressful stimuli activate the HPA axis and increase corticosterone levels, which leads to the activation of the amygdala to express the fear response and to form fear memory. Through this process, rat infants are prepared to act independently and to explore the outside world while avoiding various risks.

Similarly, in human infants, the amygdala may be involved in the formation of emotional attachment to the mother. Using the Strange Situation Procedure (Ainsworth et al., 1978), which assesses infants' behavior in the presence and absence of a caregiver and a stranger, infants who show a disturbed attachment toward their parent had a larger left amygdala volume in adulthood (Lyons-Ruth et al., 2016). The connectivity of the left amygdala to the bilateral anterior insular cortex and ventral striatum at 1 month of age has been shown to predict higher levels of fear at 6 months of age (Graham et al., 2016). Stronger amygdala connectivity to the anterior cingulate/anterior medial prefrontal cortex at 1 month of age predicts higher fearfulness, along with progress in cognitive development at 6 months of age (Graham et al., 2016).

## Neutral sensory processing during hugging

In daily life, most physical contacts between the infant and the caregiver are neither affective nor noxious but rather neutral sensations that are mainly detected via Aβ-LTMRs and Aδ-LTMRs (Figure 1E). When an infant is hugged, the infant feels a pleasant or neutral pressure that is detected through afferents in the skin and deep tissues such as subcutaneous and muscle tissues (Case et al., 2020; Mense, 2019).

**Figure 3:**
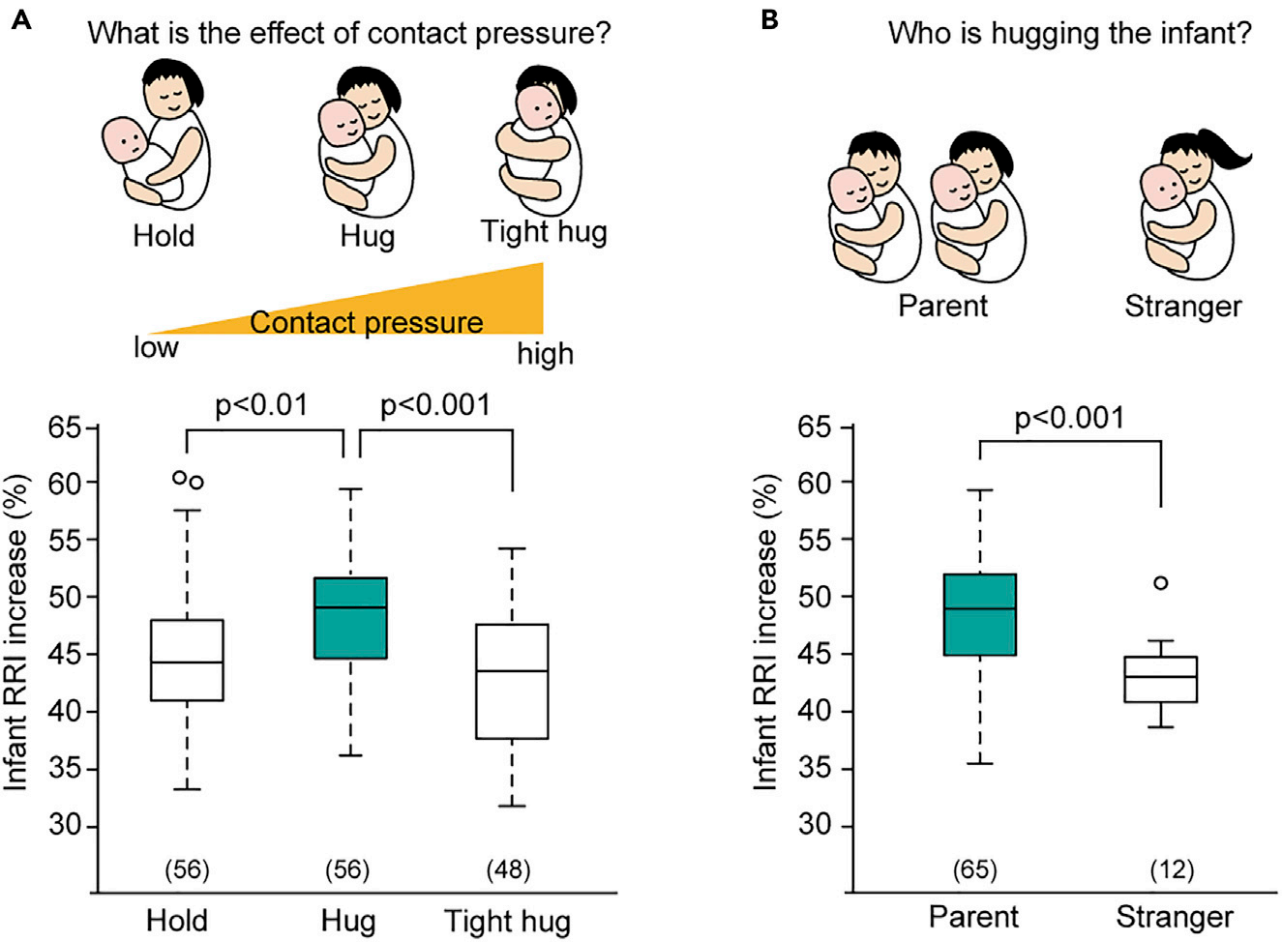
Heart rate variability during a hug in infants older than 4 months of age (A) When infants were held, hugged, or hugged tightly by their mothers, the increase in infant R-R interval (RRI), an indicator of parasympathetic activity, was greater when the infants were hugged than when they were held or hugged tightly (lower graph). The contact pressure of the mother's hand on the infant's back was highest during the tight hug among three hug/hold styles (upper scheme). (B) Infants showed a higher RRI increase when hugged by a parent than held by a stranger. Numbers in parentheses indicate the number of infants. This figure was modified from (Yoshida et al., 2020).

For example, a caregiver lifts, holds, and carries his or her infant to move, feed, clean, dress, bathe him or her, and change his or her diaper. Although similar in appearance to these carrying behaviors, caregivers often hug their infant along with positive emotions such as joy, love, happiness, and warmth toward the infant (Figure 2A). Can infants tell the difference between being held and being hugged? How does the infant feel when being hugged? As this question could not be assessed by facial expressions or verbal cues, we recorded an electrocardiogram during a certain sequence of hug/hold sessions and examined time- and frequency-domain heart rate parameters (Yoshida et al., 2020). The participants were instructed to perform three behavioral tasks for 20 s each: to hold the infant lightly as usual (hold); to hug the infant while thinking that the infant is adorable, not mechanically (hug); and to hold the infant very tightly as if the mother could run fast while holding the infant (tight hug). As a result, the R-R interval (RRI) increase, which is an indicator of parasympathetic activity in infants older than 4 months, was highest during hugging among the three hug/hold tasks (Figure 3A). The infant heart rate response was U-shaped in terms of the contract pressure between the infant's back and the mother's hand, because the pressure was higher in the order of holding, hugging, and tight hugging. Because olfactory, visual, and auditory conditions were constant during these three tasks, the only difference was the tactile sensation. The RRI increase was higher during a hug from the parents than that from a female stranger (Figure 3B), which suggests that infants' autonomic responses are dependent on their social context (Yoshida et al., 2020). Moreover, it was shown that infants recognize and prefer both their mother's and father's hugs, as the physical pressure on the infant when being hugged was different between the mother's and father's hugs. As this heart rate response was not recognized in infants younger than 4 months, social recognition, as well as autonomic nervous system maturation, are thought to be necessary. This finding is consistent with the fact that the posterior superior temporal sulcus of late infants becomes responsive to maternal touch, which suggests that the processing of somatosensory information in a social context is taking place (Brauer et al., 2016).

Infant's response to a parental hug is thought to involve many brain regions, including the insular cortex, anterior cingulate cortex, prefrontal cortex, superior temporal sulcus, amygdala, hippocampus, primary motor cortex, and cerebellum (Figures 2B–2E). These brain regions allow the infant to process a variety of somatosensory information, referring to other sensory stimuli, affective valence, social context, and memories. Then, the output pathway would include the nucleus ambiguus and dorsal vagal motor nucleus containing parasympathetic preganglionic neurons, which lead to calm the autonomic nervous system. Importantly, the caregivers also felt positive emotions and increased parasympathetic activity when they hugged their infants. Thus, the parental hug serves as a form of communication to convey feelings between the parent and the prelinguistic infant.

## Cognitive development as a basis for infant response to physical contact

Although infants have a mature sense of touch from an early age, the development of their cognitive abilities during the first year of life changes the way infants respond to physical contact the caregivers. During the first year, the structure and function of the brain drastically changes. The brain weight increases from 400 to 1,000 g, which is accompanied by increased synaptic density with the elimination of excess synaptic connections (Holland et al., 2014; Knickmeyer et al., 2008; Shaw et al., 2008). Children achieve growth milestones, gain autonomy, and become able to conduct interactive nonverbal communication. Infants can recognize and prefer a primary caregiver as early as the first month of life via olfactory, visual, and acoustic cues (Shultz et al., 2018).

Neonates may have a rudimentary perception of their own body (Filippetti et al., 2013), and the body image is developed and remapped during the first year of life (Marshall and Meltzoff, 2015). At least 3 months after birth, infants have an integrated sense of their own body with multiple perceptual modalities, including tactile, visual, auditory, and proprioceptive modalities, and they embody the sense of agency and autonomy. In parallel, infants can recognize other people, such as caregivers, as distinct entities (Rochat, 2010). Four-month-old infants can identify a new face after becoming familiar with two faces when they are gently stroked by their parents compared with when they are not stroked or stroked with a brush (Della Longa et al., 2019), indicating that pleasant tactile stimuli enhance social recognition in infants. From approximately 5 months of age, infants can distinguish between the purposeful and unintentional behaviors of other people (Woodward, 1999). Infants can comprehend several words that their parents speak as early as 6 to 9 months (Bergelson and Swingley, 2012) and begin to grasp others' intentions through spoken words. Infants follow the gazes of others from the age of 10 months (Mundy and Newell, 2007). Twelve-month-old infants can express a declarative motive to share their attention and interest with an adult experimenter by pointing to a flying toy (Liszkowski et al., 2004). The pointing duration and number change depending on whether the experimenter also pays attention to the toy. Infants begin to read the mind of others from approximately 12 months of age when they communicate with others in terms of comprehension and attention (Liszkowski, 2014; Tomasello et al., 2007). Thus, by 12 months of age at the latest, infants are able to perform joint attention to coordinate their attention with their social partners (Mundy and Newell, 2007). When 12-month-old infants see a person who is looking for something, they read the intention and help them by pointing and providing the necessary information (Liszkowski et al., 2008).

In the first year of life, children learn the boundaries between “me” and “not me” through somatosensory perceptions of themselves and their caregivers; through physical and nonphysical interactions with their primary caregivers, they form the foundation for exploring the outside world and building emotional and social relationships with others in the future. As human adults communicate emotion through physical contact (Gallace and Spence, 2010), infants can communicate nonverbally with caregivers about their feelings. In fact, primitive communication between prelinguistic infants and caregivers is mostly mediated through physical interaction.

Consistent with the fact that physical interaction with the mother is the primary source of somatosensory and other sensory stimuli, the presence of the mother and certain types of maternal care behavior can alter the local field potential (LFP) of the cerebral cortex in infant rats (Sarro et al., 2014). The absence of the mother increases desynchrony with increased beta and gamma frequency power, whereas nipple attachment decreases desynchrony and increased slow-wave activity (Sarro et al., 2014). The presence of the mother increases the delta range (0–4 Hz) power in the LFP of the anterior cingulate cortex of infant rats. Serotonin [5-hydroxytryptamine] 2 receptor antagonist can block this effect (Courtiol et al., 2018), which suggests that serotonergic transmission is involved in the enhanced low frequency in response to maternal interaction. Importantly, adversity rearing by the mother that is induced by a low level of bedding material results in blunted LFP responses to grooming and milk ejection by the mother (Opendak et al., 2020). These findings show that the proximity and physical contact of caregivers alter brain states and have a relaxing effect on infants.

## Developmental changes in sensorimotor reflexes to being carried

**Figure 4:**
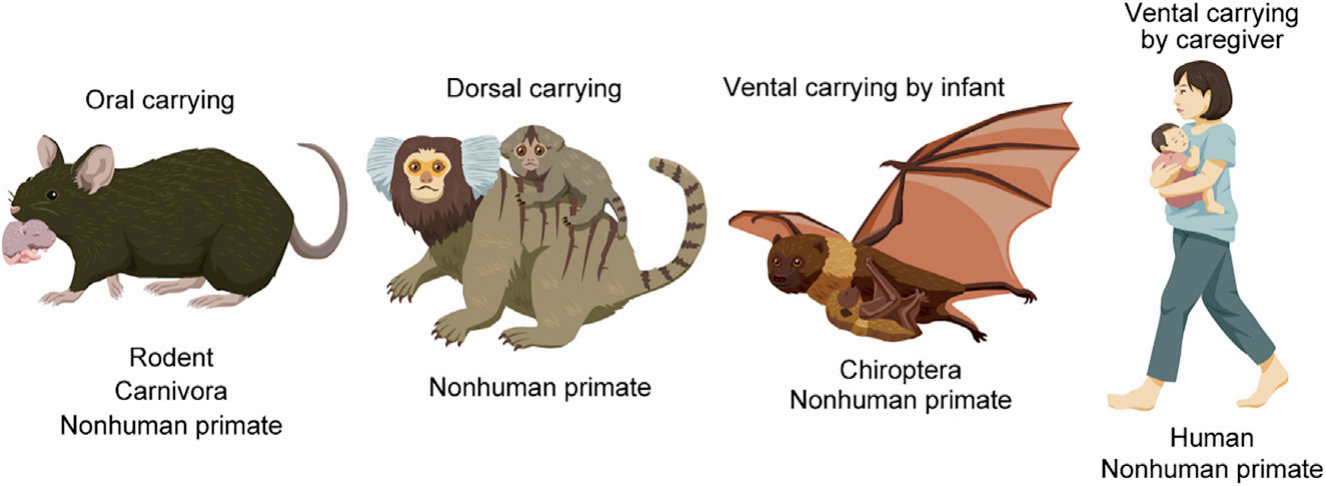
Mammalian infant carrying Oral carrying (rodents, carnivores, primates): a mother animal holds the infant in her mouth and moves around. Dorsal carrying (primates): an infant clings to the back of a moving mother. Ventral carrying (bats, primates, humans): an infant clings to the front fur of a moving mother or is held by the mother by the hands.

Newborn altricial mammals with immature motor ability need caregivers, usually, mothers to move, to take care of, and to feed them. Caregivers carry their children in many different styles (Figure 4). Quadrupedal mammals such as rodents and carnivores carry their infants by oral grasping (Esposito et al., 2013; Lezama-García et al., 2019). Dorsal carrying is observed in nonhuman primates whose infants grab the fur on the mother's back. Some people carry their infants on their backs while using tools. Ventral carrying without caregiver support is observed in bats and nonhuman primates whose newborns have enough clinging ability. Primates, including humans, use their upper limbs to carry their infants ventrally (Berecz et al., 2020; Ross, 2001).

What all carrying styles have in common is that there is physical contact and that the infant is calm while being carried. When you are raising an infant, you may notice that as soon as you walk around with a crying infant in your arms, the infant immediately stops crying and calms down; however, when you stop carrying the infant, the infant starts crying again. Real-time quantitative analyses have revealed that in infants younger than 6 months, being held by their mothers while walking results in an immediate reduction in crying, voluntary movements, and heart rates (Esposito et al., 2013).

**Figure 5:**
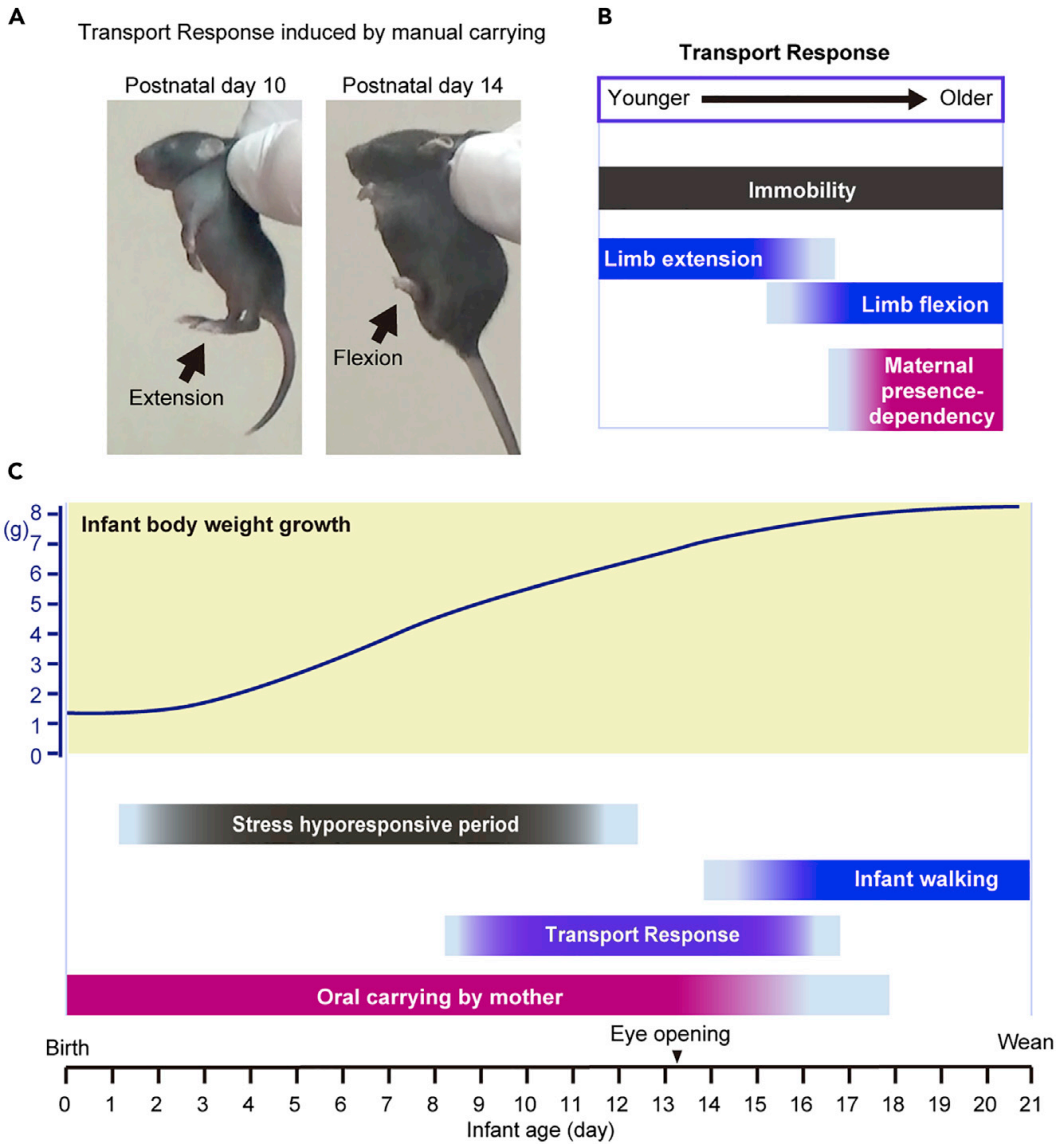
Transport Response in mouse infant (A) Manual carrying using experimenter's fingers induced Transport Response. Both 10-day-old and 14-day-old infants showed immobility. The 10-day-old infant showed lower limb extension (left), whereas the 14-day-old infant showed lower limb flexion (right). (B) Immobility is an essential component of Transport Response. As the infant grows, the posture of the lower limb changes from extension to flexion. In an older infant, the occurrence and maintenance of Transport Response depend on the presence of the mother. (C) Timeline of mouse infant from birth to weaning. The upper graph indicates infant body weight growth. Transport Response occurs during the transition from oral carrying by mother to walking on their own (Esposito et al., 2013; Yoshida et al, 2013, 2018).

When a mother mouse holds her infant with the scruff of its neck in her mouth, the infant exhibits a certain set of behaviors, which is called Transport Response. Transport Response consists of a decrease in ultrasonic vocalization, the cessation of voluntary movements in a characteristic compact posture with the flexed limbs, a decrease in heart rates, and an increase in the threshold for pain sensation (Esposito et al., 2013; Yoshida et al., 2013). Transport Response can be induced when an experimenter plucks the back of the infant's neck with his or her fingers and lifts it (Figures 5A and 5B). Thus, although the mouse Transport Response is not a typical attachment behavior that requires highly selective bonding between the infant and the caregiver, a coordinated motor and autonomic response to somatosensory and proprioceptive stimuli promotes proximity to the caregiver. As local anesthesia with lidocaine suppressed Transport Response (Esposito et al., 2013), the tactile sensation via Aβ-, Aδ-, and C-sensory fibers may be involved in eliciting the carrying-induced calming response. The motor response to pinching a tail with a small clip was suppressed during Transport Response, indicating an analgesic effect (Yoshida et al., 2013). The cerebellar cortex is involved in postural regulation during Transport Response (Esposito et al., 2013).

Mouse pups exhibit Transport Response from postnatal day 8 to days 16–18, which helps mothers safely carry their growing infants by mouth (Figure 5C). In response to manual carrying by the experimenter, Transport Response usually begins to appear around postnatal day 8, and the pup becomes immobile and slightly flexes its upper and lower limbs. Before this day, the mouse pups are small and light enough to be easily carried. In addition, postnatal day 8, is in the middle of the stress hyporesponsive period, which lasts until approximately postnatal day 12 (Schmidt, 2019). After the end of the stress hyporesponsive period at postnatal day 14, the expression of Transport Response becomes increasingly social context dependent. Transport Response was suppressed when the mother was not present in the home cage or when the pup was alone in the novel environment (Figure 5B) (Yoshida et al., 2018). The activation of the anterior cingulate cortex that is due to corticotropin-releasing hormone from the paraventricular nucleus is thought to underlie this social context-dependent suppression of Transport Response (Yoshida et al., 2018). From postnatal days 14–18, the duration of Transport Response decreases, but the hindlimbs are fully flexed during the Transport response. After postnatal day 18, mouse infants can move on their own and do not express Transport Response when plucked on the back by the mother or an experimenter. Thus, the initiation and cessation of Transport Response seems to be designed to correspond to the pup's sensory-motor, neuroendocrine, and social development and to allow the pup to be independent of the mother.

## Long-term effect of lack of appropriate physical contact in infancy

Social deprivation during infancy has been well reported to have adverse and long-term effects on physical, intellectual, and mental growth, with social difficulties (Bakwin, 1942; Rutter et al., 2007). Social deprivation affects all sensory modalities, but a pediatrician named Bakwin found that the introduction of infant care with physical contact by nurses and interns and the acceptance of parental visits drastically reduced infant mortality (Bakwin, 1942).

A cohort study of adverse experiences is being conducted with children who were abandoned and grew up in an institution in Bucharest, Romania, and then either were randomly fostered in the United Kingdom or remained in the institution (Nelson et al., 2007; Rutter et al., 2007; Zeanah et al., 2017). In the Romanian orphanage, children were severely socially deprived. After adoption by UK families, the children showed marked psychological and physical catch-ups at the ages of 6 and 11 years; however, a minor population of children who were adopted after the age of 6 months continued to have cognitive deficits, autistic features, emotional and conduct disturbances, and disinhibited attachment (Rutter et al., 2007). Despite years of environmental enrichment in adoptive homes, these children showed smaller total brain volume compared with adoptees who had not lived in Romanian orphanages during infancy (Mackes et al., 2020). The extent of the volume reduction was correlated with the duration of institutionalization. The reduction in brain size was also associated with a lower intelligence quotient and elevated attention-deficit/hyperactivity disorder (ADHD) symptoms in fostered individuals. Thus, severe deprivation in the first years of life is associated with changes in adult brain structure, despite normal family life thereafter. An adverse family environment causes growth retardation through reversible hypopituitarism (Powell et al., 1967; Rogol, 2020). Currently, psychosocial short stature (ICD11, 5B11) is used to describe children who are raised under adverse conditions and show growth failure characterized by suppressed height, weight, and head circumference, in which growth hormone and insulin-like growth factor 1 are thought to be involved (Johnson and Gunnar, 2011).

Harlow showed that total social deprivation of rhesus monkey infants for the first 6 months of life caused severe deficits in social behavior such as sexual behavior and parental behavior when they grew up (Harlow and Suomi, 1971). Similarly, artificially reared female rats showed significantly less licking and crouching behaviors toward their pups in their own parenting (Gonzalez et al., 2001). Conversely, when bonobos, our closet primate relatives, are reared by mothers, the juvenile bonobos are more likely to show consolation behaviors and to have high empathy compared with orphaned bonobos (Clay and de Waal, 2013). These findings suggest that physical contact with a caregiver with positive emotional valence in infancy modifies functional neural connectivity and develops the neural basis of empathy behavior and proper social behavior in adulthood.

In addition to social, emotional, and physical development, contact experiences during infancy may be required to form pleasant sensations from the skin and deep tissues. A foster cared for group of young adults who had a high tendency of neglected and/or abusive experience in their childhood displayed lower pleasant scores for gentle stroking than did an age-matched control group, which suggests the involvement of the early experience of gentle stroking to foster pleasant sensations in later life (Devine et al., 2020). As being hugged causes a deep pressure sensation in infants, parental hugs in early life may foster pleasant deep pressure in later life (Case et al., 2020). Insensitivity to pleasantness during physical contact with others may interfere with the development of intimate relationships with others in adulthood. It is possible that there is a critical period for the development of tactile processing with positive valence in a context-dependent manner. Interestingly, ligand binding of oxytocin receptors in the cerebral cortex is highest during infancy in mice (Hammock and Levitt, 2013), suggesting the possibility that oxytocin system links social deprivation to inappropriate maturation of somatosensory system.

Maternal separation from infants has been used to study early life stress mainly in rodents, as it leads to depression after maturation and an altered HPA axis (Glover et al., 2010; Millstein and Holmes, 2007; Nishi et al., 2014; Tractenberg et al., 2016). However, early tactile intervention can reduce the impact of adverse experiences in infancy. Brush strokes that mimic maternal licking are given to artificially reared infant rats twice a day, which alters the anxiety behaviors of the rats after weaning (Gonzalez et al., 2001). Thus, consistent with the Kangaroo care, somatosensory stimulation during infancy partially compensates for the deprivation of maternal physical contact.

## Physical contact with infants can change caregivers

The classical Harlow experiment examined how physical contact from an infant changes the parental behavior of motherless mothers (Harlow and Suomi, 1971). Harlow found that motherless mother monkeys who have not experienced any care from a mother because of total social deprivation from birth either ignore or abuse their initial offspring. However, as the infant monkey keeps asking for physical contact from the motherless mother for several months, the mother monkey finally allows physical contact and decreases her brutal behaviors toward the infant. Subsequently, the mother monkeys behave better toward the second or third child (Harlow and Suomi, 1971). Thus, physical contact between the mother and the infant is not unidirectional but reciprocal, and it can alter the behavior and mood of the mother.

Oxytocin may help form bonds through physical contact between the infant and the parent. The baseline levels of plasma and salivary oxytocin in the mothers and fathers who have infants aged 4 to 6 months are similar, whereas after 15 min of free “play and touch” interaction with the infant, salivary oxytocin levels increases in mothers and fathers with more contact but not in mothers and fathers with less contact (Feldman et al., 2010). Similarly, when the parent hugs their infant, the parent exhibits an increase in heart rate interval, which indicates parasympathetic activity and more secure feelings (Yoshida et al., 2020). Thus, physical interaction with one's infant leads to psychological, autonomic, and neuroendocrine changes in the caregiver.

## Future direction

There is no doubt that physical contact with the caregiver is important for the proper development of infants and that it has long-term effects. However, most studies have been observational studies using subjective parameters; the few studies using physiological and objective parameters have only been conducted in a laboratory. Advances in sensor technology will allow us to examine physical contact between the infant and the caregiver in a more natural and everyday living environment. This approach will answer many questions, for example, whether there are gender differences in the effects of infant physical contact in adulthood, its impact on longevity, and the interaction between genes and the environment. We believe that proper understanding can free parents and society from the myths surrounding parenting. Noninvasive long-term recording of electroencephalograms will answer whether co-sleeping, which increases physical contact between the infant and the caregiver, has any benefit on sleep quality, daily activity, and psychophysical development (Sadeh et al., 2010).

In contrast to the rapid progress in our understanding of cutaneous sensory afferents, there is very little research on afferents from deep tissues. However, given that various massage therapies target not only the skin but also deeper tissues, the afferents of muscle and subcutaneous tissue afferent may play an important role in the perception of pleasant somatic sensations and relaxation during massage.

Research aimed at understanding autism spectrum disorder (ASD) is also needed. Children with ASD often show repetitive sensory-motor behavior (Lord et al., 2018; Shultz et al., 2018). They exhibit hypersensitivity to sensory stimuli that leads to defensive responses and hyposensitivity to tactile stimuli that leads to the repetition of certain sensations, such as the repetitive brushing of the skin (Cascio, 2010), which indicates abnormal somatosensory perception in ASD. Although ASD is rarely diagnosed during the first year of life, altered social communication with caregivers can be retrospectively seen as early as the later part of the first year (Elsabbagh and Johnson, 2010; Wan et al., 2019), when early signs of morphological brain development differences, such as the hyperexpansion of the cortical surface area were observed in infants who were later diagnosed with autism (Hazlett et al., 2017). Anecdotally, the first report of autism by Kanner mentioned that almost all mothers were surprised that their infants did not assume the anticipatory postural preparation for being picked up (Kanner, 1943). Thus, a longitudinal study of infant-caregiver interactions in daily life can identify early markers of ASD and develop early interventions to enhance social and interpersonal development.

The beneficial effects of physical contact can be used to develop mechanical devices to reduce social stress and physical pain for children in institutions, orphanages, hospitals, or any other situation in which they are in need (Eckstein et al., 2020). These effects can ease the amount of distress in adults who suffer from social pain and loneliness in an aging and individualistic society.
